# Exploring Nutrient Profiles, Phytochemical Composition, and the Antiproliferative Activity of *Ganoderma lucidum* and *Ganoderma leucocontextum*: A Comprehensive Comparative Study

**DOI:** 10.3390/foods13040614

**Published:** 2024-02-18

**Authors:** Guoqin Peng, Chuan Xiong, Xianfu Zeng, Ya Jin, Wenli Huang

**Affiliations:** 1 Biotechnology and Nuclear Technology Research Institute, Sichuan Academy of Agricultural Sciences, Chengdu 610061, China; 18784370622@139.com (G.P.);; 2 School of Food and Biological Engineering, Chengdu University, Chengdu 610106, China; 3 Chengdu Academy of Agriculture and Forestry Sciences, Chengdu 611130, China

**Keywords:** *G. lucidum*, *G. leucocontextum*, chemical composition, antioxidant, antiproliferation

## Abstract

*Ganoderma*, often hailed as a holistic “health package”, comprises an array of nutritional components and active compounds, contributing to its esteemed status in the realm of healthy foods. In this study, a comprehensive analysis was performed to elucidate the diverse nutritional profiles, bioactive components, and antiproliferative activities between two *Ganoderma* species: *G. lucidum* (GLU) and *G. leucocontextum* (GLE). The results showed that GLE possessed a higher level of nutritional constituents, except for dietary fiber. Fatty acid analysis revealed comparable profiles rich in unsaturated fatty acids for both species. The ethanol extract of GLU and GLE exhibited potent antioxidant capabilities and remarkable inhibition of tumor cell proliferation via apoptosis induction, with greater potency in GLE. The heightened triterpene levels in GLE potentially contribute to its augmented antitumoral effects. The exploration emphasized the significance of comprehending the varied chemical compositions of *Ganoderma* species, providing insights into their potential health benefits applications in the food and pharmaceutical industries.

## 1. Introduction

*Ganoderma* spp., commonly known as “Reishi” in Japan or “Lingzhi” in China, is a remarkable genus of macrofungi with an extensive history of traditional usage both as functional sustenance and as a therapeutic agent. To date, in excess of 428 distinct species of *Ganoderma* have been identified [1]. A burgeoning focus in the scientific research has accentuated its medicinal attributes. *Ganoderma* was historically employed as a remedial measure for an array of health considerations, embodying its potential as a source of vitality, longevity, and overall well-being. Research initiatives have unveiled a myriad of noteworthy qualities exhibited by *Ganoderma*, including immune system enhancement, antibacterial, antitumor, anti-inflammatory, lipid-reducing, antiatherogenic, antifungal, and antiviral properties [2]. Their preeminent contributions to health benefits emanate from their nutritional composition and bioactive constituents, encompassing polysaccharides, triterpenes, protein, sterols, and other secondary metabolites [3]. Over 450 secondary metabolites from *Ganoderma* have demonstrated remarkable therapeutic properties [4]. Nevertheless, the nutritional composition and active ingredients differ among various *Ganoderma* species, giving rise to consequential distinctions in bioactivity [5]. Recently, a comparative analysis was conducted on the chemical composition and antioxidant and antiproliferative activities of diverse *Ganoderma* collections. The findings indicated a close correlation between these abilities and the active ingredients [6]. Mohammadifar et al. discovered that the total phenols, flavonoids, and betulinic acid in *Ganoderma applanatum*, along with its antioxidant activity, were higher than in the other species. On the other hand, *G. lucidum* showed higher levels of total polysaccharides and proteins [7]. The contents of β-glucans, ergosterol, and vitamin D2 varied among the different *Ganoderma* species [8]. Notably, polysaccharides and triterpenes, particularly ganoderic acid, emerged as the pivotal active components, predominantly responsible for their therapeutic properties [9]. The content of polysaccharides and triterpenes serves as a crucial indicator in evaluating the quality of *Ganoderma*. Analyzing the chemical composition among *Ganoderma* spp. is not only pivotal for quality assessment but also conducive to elucidating their pharmacological activities.

Among *Ganoderma* species, *Ganoderma lucidum* stands out as the most well-known and widely distributed species in Asia. It has been extensively studied for its multifaceted bioactivities in the fruiting bodies, mycelia, and spores, including antioxidative, antiaging, antitumor, anti-inflammatory, immune-regulatory, neuroprotective, anti-HIV, and hypoglycemic properties [2,10]. *G. lucidum* is not only harnessed as a medicinal remedy but also for its broad applications across various industries, such as nutraceuticals, health supplements, and cosmetics [11]. Proximates, minerals, and a diverse array of bioactive compounds in *G. lucidum* have undergone extensive scrutiny and are comprehensively understood [12,13]. In recent years, *Ganoderma leucocontextum*, a newly discovered species from Tibet plateau and Sichuan Province in China, has garnered attention [14]. *G. leucocontextum* shows promise in neuroprotection, neurite growth promotion, HMG-CoA reductase inhibition, and antioxidant activity attributed to its polysaccharides and triterpenes [15,16,17]. Moreover, *G. leucocontextum* demonstrates potential in cancer treatment by inducing tumor cell death via mitochondrial dysfunction targeting lipid metabolism [18]. However, limited data regarding the nutritional value and chemical composition of *G. leucocontextum* are currently available.

Therefore, the research aims to conduct a comparative analysis of the nutrition profile and phytochemical composition between two *Ganoderma* species, *G. lucidum* and *G. leucocontextum*. Additionally, the antioxidant capacity and the ability to inhibit tumor cell growth were investigated to assess their bioactivity, thereby contributing to a more comprehensive understanding of their potential health benefits and further exploration of their applications in food and medicine.

## 2. Materials and Methods

### 2.1. Mushroom Species

The *G. lucidum* strain was donated by Sichuan Wanzhi Biological Technology Co., Ltd. (Chengdu, China). The *G. leucocontextum* strain was isolated from Kangding, Sichuan, China, and preserved at Chengdu Academy of Agriculture and Forestry Sciences. Molecular characterization of both strains was carried out by sequencing based on the internal transcribed spacer region (ITS) of ribosomal DNA. These two original strains were used to prepare the spawn via a three-step operation. Briefly, mycelium revitalization was performed with a PDA medium in test tubes. Then, the spawn attained large-scale production through sub-culturing in a new sterilized media (70% sawdust, 20% corn cob, 10% bran). Once the mycelium had colonized all the material, the spawns were individually inoculated into autoclaved substrates to induce the formation of fruiting bodies. The fruiting bodies of *G. lucidum* and *G. leucocontextum* were harvested at the mature stage with the disappearance of the white growth edge. All samples were dried in the oven and further pulverized. Voucher specimens of these samples were deposited in the freezer in Biotechnology and Nuclear Technology Research Institute, Sichuan Academy of Agricultural Sciences.

### 2.2. Cell Lines and Chemicals

Rat adrenal pheochromocytoma cells (PC12), mouse melanoma B16 cells, human cervical carcinoma cells (Hela), and human pulmonary carcinoma cell (A549) lines were sourced from ATCC, Manassas, VA, USA. Cell culture supplies (Dulbecco’s Modified Eagle’s Medium, fetal bovine serum, trypsin-EDTA, streptomycin–penicillin) were acquired from Gibco (Grand Island, NY, USA). The standard mixture of 37 Fatty Acid Methyl Esters (FAMEs) was obtained from Supelco (Bellefonte, PA, USA). Standard quercetin, gallic acid, and oleanolic acid with a purity of more than 98% were purchased from Shanghai YuanYe Biotechnology (Shanghai, China). Fluorescent dye and CCK-8 assay kit were purchased from Shanghai Biyuntian Biological Co., Ltd. (Shanghai, China). Other reagents and chemicals used in the experiments were of analytical grade.

### 2.3. Proximate and Mineral Analysis

The dry fruiting bodies were chopped and powdered before determination of their components. The chemical composition of *Ganoderma*, including moisture content, ash content, crude fiber, crude protein, gross fat, and mineral content, was analyzed following Association of Official Analytical Chemists (AOAC) procedures [19]. To measure the moisture content, samples were dried in an oven at 133 °C until a constant weight was achieved. The loss of mass was measured to calculate the moisture content. Subsequently, the dry samples underwent incineration in a muffle furnace at 600 ± 15 °C until complete combustion, leaving only inorganic ash. The gross fat of samples was obtained through Soxhlet extraction with petroleum ether. After the ether has evaporated, lipid residue was weighed to calculate the content of gross fat. For the determination of crude fiber, the samples were subjected to the removal of fat and non-fibrous components using an acid-alkali mixture. The resulting insoluble fiber residue was washed, filtered, dried, and weighed. The crude fiber content was calculated as a percentage of the original sample weight. The protein content was estimated using the Kjeldahl method determined with distillation unit (Gerhardt, Germany) [20]. Levels of minerals were determined using an Agilent 240 FS flame atomic absorption spectrophotometer (Agilent Technologies, Santa Clara, CA, USA) after calibrating the equipment with appropriate standard solutions.

### 2.4. Analysis of Amino Acids

A total of 1 g of powder was thoroughly mixed with 15 mL of 6 M HCl containing 0.5% phenol in a digestion tube. The tube was then injected with nitrogen and hydrolyzed in a drying oven at 110 °C for 22 h. The hydrolysate was lyophilized and dissolved in 2 mL of sodium citrate buffer at pH 2.2. Prior to analysis using an amino acid analyzer, the solution was filtered through a 0.22 μm membrane. The amino acid profile was determined using an automated amino acid analyzer through ion-exchange chromatography with post-column derivatization using ninhydrin.

### 2.5. Fatty Acid Analysis

For fatty acid analysis, the samples underwent ultrasonic extraction with methanol—chloroform (1:4, *v*:*v*) mixture at 140 W for 20 min, repeating the process three times. The extracts were methylated to fatty acid methyl esters according to Tokul-Olmez [21]. GC-MS analysis was employed for determining fatty acid methyl esters, utilizing a standard mixture of 37 fatty acid methyl esters for instrumental parameter optimization. The optimal injector temperature was set at 250 °C, and a temperature ramp was used to increase the column temperature from 40 °C to 230 °C. The initial column temperature was maintained at 40 °C for 3 min then increased to 180 °C at a rate of 5 °C/min and held for 5 min. Then, it was raised to 220 °C at a rate of 4 °C/min and maintained for 5 min. Finally, the temperature was increased to 230 °C at a rate of 4 °C/min for 10 min. The quadrupole mass filter was maintained at 250 °C and programmed in the scan mode from 45 to 550 *m*/*z* with a frequency of 3.9 Hz.

### 2.6. Determination of Total Phenol

The determination of total phenol was performed according to Sidhu et al. with some modification [22]. A mixture of 1 g of powder and 40 mL of anhydrous ethanol were sonicated for 25 min at 240 W. The filtrate was adjusted to a volume of 50 mL in a volumetric flask. Subsequently, 1 mL of the filtrate solution was mixed with 9 mL of distilled water and 0.5 mL of Folin–Ciocalteu’s phenol reagent. The mixture underwent a reaction at room temperature for 6 min. A mixture of 5 mL of 5% Na_2_CO_3_ solution was rapidly added and mixed thoroughly. Then, the mixture was diluted to a total volume of 25 mL with distilled water and incubated for 90 min at room temperature in a light-free environment. Finally, the optical density was measured using a spectrophotometer at a wavelength of 750 nm. Gallic acid (GA) was employed as a standard to calculate the content of total phenol based on the calibration curve (Y = 0.0018X + 0.0578; R^2^ = 0.9999). The total flavonoids content was expressed as mg gallic acid equivalent (GAE)/g dry weight (D.W.).

### 2.7. Determination of Total Flavonoids

The sample was prepared as described above. Total flavonoid content was calculated using the method developed by Mohammadifar et al. [7]. A total of 1 mL of the resulting filtrate was mixed with 2 mL of distilled water and 150 μL of 5% NaNO_2_ for 5 min. Following this, 150 μL of 10% AlCl_3_ was introduced and the reaction was allowed to continue for an additional 5 min. Then, 1 mL of 1 M NaOH was added to react for 30 min. Quantification was performed by comparing the optical density of quercetin (QE) measured with a spectrophotometer at 415 nm. The total flavonoids content was calculated from the calibration curve of quercetin (Y = 0.0036X + 0.0716; R^2^ = 0.9987) and expressed as mg quercetin equivalent (QE)/g dry weight (D.W.).

### 2.8. Determination of Triterpenes

Ultrasonic generators were used to assist in triterpenoids extraction with anhydrous ethanol. The yield of total triterpenoid was determined by the vanillin–glacial acetic acid–perchloric acid method [23]. Briefly, the extract of triterpenoid was incubated with perchloric acid and 5% vanillin–glacial acetic acid for 15 min in a water bath at 70 °C, followed by the addition of ethyl acetate. After cooling to room temperature, the absorbance of the mixture was measured at 545 nm using a spectrophotometer. Oleanolic acid was employed to create a standard curve.

### 2.9. Analysis of Polysaccharide

The polysaccharide of *Ganoderma* was prepared via hot water extraction, alcohol precipitation, and deproteinization as described previously [24]. The powder was initially soaked in anhydrous ethanol for 24 h to remove fat. After evaporating the ethanol, the residue was added to 20 times its volume of distilled water and heated in a 95 °C water bath for 6 h, repeating three times. The supernatants were collected and concentrated. The concentrate was mixed with 9 times its volume of anhydrous ethanol for precipitation, which was allowed to stand for 24 h. After removing the protein with Sevage reagent (chloroform–n-butanol = 4:1, *v*:*v*), the crude polysaccharide was lyophilized. The content of polysaccharide was determined via the anthrone–sulfuric acid method.

### 2.10. Antioxidant Activity Assay

The ethanol extracts of *G. lucidum* (GLUT) and *G. leucocontextum* (GLET) were subjected to screening for antioxidant activity via DPPH, ABTS free-radical-scavenging activity, and Fe^3+^ reducing power (FRAP) assay.

#### 2.10.1. DPPH Radical Scavenging Activity

Antioxidant activity was evaluated via DPPH solution to measure the free-radical-scavenging activity of the extracts [25]. A solution of 150 μL 0.12 mmol/L DPPH was mixed with 150 μL of triterpene at various concentrations, with anhydrous ethanol as a control. After reaction for 15 min, the absorbance was measured at 517 nm. The calculation formula for the DPPH free radical scavenging rate was as follows:

Scavenging activity (%) = (A_control_ − A_sample_)/A_control_ × 100.

#### 2.10.2. ABTS Radical Scavenging Activity

According to the method developed by Zhang et al. [26], a reaction mixture of 200 μL of the diluted ABTS cationic radical solution and 50 μL of triterpene solution at varying concentrations was prepared. Anhydrous ethanol was used as the Control. After reacting at room temperature for 15 min, the absorbance was measured at 734 nm. The clearance rate was calculated using the following formula:

Scavenging activity (%) = (1 − A_sample_/A_control_) × 100.

#### 2.10.3. Reducing Power (Fe^3+^) Assay

The determination of reducing power was performed following the method of Li et al. with some modification [27]. A total of 0.2 mL of triterpene solution with various concentrations was combined with 0.2 mL phosphate buffer salt, along with 0.5 mM of potassium ferricyanide. After incubation at 50 °C for 20 min, 2.5 mL of 10% trichloroacetic acid was added to halt the reaction. The supernatant was mixed with an equal volume of distilled water; then, 0.5 mL of 0.1% FeCl_3_ was added. The absorbance of the mixture was measured at 700 nm.

### 2.11. Determination of Cell Viability

PC12, B16, Hela, and A549 cells were maintained as adherent monolayers in Dulbecco’s modified Eagle medium (DME) supplemented with 10% FBS and 1% streptomycin/penicillin in a humidified incubator at 37 °C with 5% CO_2_. The Cell Counting Kit-8 (CCK-8) assay was used to determine cell viability following the instructions provided by the manufacturer. Cells in the logarithmic phase were seeded into 96-well plates at a density of 1 × 10^4^ cells/well. After incubation for 24 h, the cells were treated with the extracts at concentrations ranging from 7.8125 to 500 μg/mL for 48 h. Subsequently, 10 μL of CCK-8 solution was added to each well for an additional 4 h. Cell viability was determined by measuring absorbance at 450 nm using a multifunctional microplate reader. The cell viability was presented as a percentage using the following formula: cell viability (%) = (A_sample 450_ − A_blank_)/(A_control 450_ − A_blank_) × 100%.

### 2.12. TUNEL Assay

TUNEL assay was employed for the detection of cell apoptosis. PC12 cells were seeded into 24-well plates at appropriate densities and incubated overnight. The cells were treated with 50 μg/mL of extract or sterile-deionized water for 24 h. After being washed twice with PBS, cells were labeled with Cy3-dUTP (red), while nuclei were counterstained by 4′,6-diamidino-2-phenylindole (blue). Then, the cells were immediately observed under a fluorescence microscope.

### 2.13. Flow Cytometric Assay

To investigate the death pathways of PC12 cells induced by extracts of *Ganoderma*, flow cytometry was employed for the apoptosis assay. PC12 cells were cultured in a 6-well plate at a density of 1 × 10^6^ cells/mL and incubated overnight. The supernatant was replaced with the fresh medium with or without 50 μg/mL extracts. After treatment for 24 h, the cells were resuspended in 1 mL cold PBS and stained with fluorescein-isothiocyanate (FITC)-conjugated Annexin V and propidium iodide (PI) for 30 min on ice in the dark. Samples stained with fluorescein-isothiocyanate (FITC)-conjugated Annexin V alone or PI alone, and unstained cells was used to set the gating and compensation for the experiment.

### 2.14. Statistical Analysis

All data were obtained from the experiments in triplicate and expressed as mean ± SD. Statistical analysis was performed using SPSS (version 19.0) and GraphPad Prism (V.5.0) software. Student’s *t*-test and one-way analysis of variance (ANOVA) was used to assess the continuous variables. Two-tailed *p* < 0.05 was considered statistically significant.

## 3. Results and Discussion

### 3.1. Proximate Analysis and Mineral Composition of GLU and GLE

*Ganoderma* is esteemed as a comprehensive “healthful ensemble”, providing a bountiful source of nutritional constituents and active compounds. Numerous investigations have underscored substantial variations in the chemical composition of *Ganoderma* across diverse origins or varieties [5]. Proximate analysis of *G. lucidum* has undergone comprehensive scrutiny, revealing properties characterized by elevated protein, low fat, and abundant dietary fiber [12]. Currently, there is a lack of available data on the nutritional value of GLE, making it challenging to compare the differences between GLU and GLE. In this study, the disparities in proximate, amino acid profile, and mineral composition between GLU and GLE are depicted in Table 1. The protein and gross fat contents in GLU were 14 ± 0.36% and 2.02 ± 0.07%, respectively. This aligned consistently with previous findings, indicating protein content ranging from 9.93% to 16.79% in *G. lucidum* [12,13,28]. Dietary fiber emerged as the predominant component in *Ganoderma*, with Fraile-Fabero et al. illustrating that dietary fiber from carpophores of *G. lucidum* constituted more than 50%, while the total gross fat content remained only 1.26% [13]. The presence of copious crude fiber and low fat holds nutritional significance in reducing cholesterol levels and promoting intestinal health [29]. GLE exhibited elevated levels of protein (21.2 ± 0.31%) and ash (2.2 ± 0.17%) compared to GLU. Conversely, GLU demonstrates a higher abundance of dietary fiber (64.2 ± 2.3%) than GLE (58.9 ± 1.3%). This implies that GLU may encompass a higher carbohydrate content.

Furthermore, a noteworthy disparity in mineral composition was observed between the two *Ganoderma* species. The magnesium (486 mg/kg), iron (38.5 mg/kg), and zinc (18.3 mg/kg) contents in GLU were lower than in GLE (750 mg/kg, 69.9 mg/kg, and 42.9 mg/kg), while the calcium (1670 mg/kg) content exceeded that of GLE by more than twofold (683 mg/kg). Our results align with prior findings for *Ganoderma* from Ghina, where major mineral elements and trace elements accounted for 430–2048 mg/kg and 5.6~2990 mg/kg, respectively [5]. The total amino acid content of GLE was 119 mg/g, whereas it was only 79.5 mg/g in GLU. As illustrated in Table 1, substantial distinctions were observed in the amino acid profiles between GLU and GLE. Except for methionine, isoleucine, and phenylalanine, the concentration of other amino acids in GLE, encompassing eight essential amino acids, surpassed that in GLU. Particularly noteworthy was the content of arginine (8.0 ± 0.4 mg/g) in GLE exceeding that in GLU by more than two-fold. The concentration of aspartic acid, glutamic acid, and histidine in GLE surpassed 10 mg/g. Our findings conclusively established that GLE contained higher quantities of protein, fat, minerals, and amino acids compared to GLU. These results suggested that GLE possessed superior nutritional value, thereby contributing to the enhancement of overall bodily function and well-being.

### 3.2. Fatty Acid Composition of GLU and GLE

**Figure 1:**
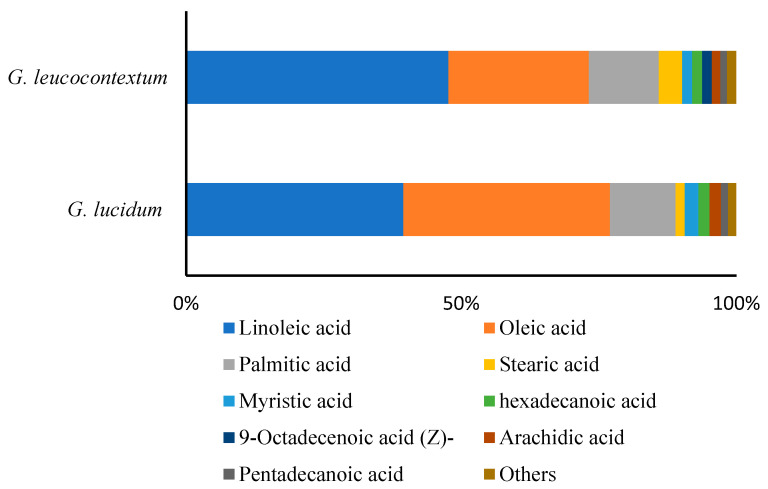
Fatty acid composition of GLU and GLE.

The fatty acid composition of GLU and GLE exhibited a high degree of similarity (Figure 1). Unsaturated fatty acids predominated over saturated fatty acids, with linoleic acid and oleic acid emerging as the primary fatty acids in *Ganoderma*, followed by palmitic acid and stearic acid, in accordance with prior investigations [8,28,30]. The combined proportion of linoleic acid and oleic acid accounted for more than 70% of the total fatty acid content in both *Ganoderma* species. Linoleic acid, constituting 39.48% in GLU and 47.67% in GLE, played a pivotal role in various physiological functions, encompassing the reduction of cardiovascular diseases, blood pressure, and the minimization of triglyceride levels [31]. This suggested that *Ganoderma*, particularly GLE, could be advocated as a component of a healthful diet for individuals with elevated cholesterol levels. Furthermore, the linoleic to oleic acid ratio, employed as a crucial criterion from a chemotaxonomic perspective, proved valuable in distinguishing among species within the same genus [32]. In this study, the ratio of linoleic to oleic acid was 1.05 for GLU and 1.87 for GLE. Lv et al. determined the fatty acid content in nineteen *G. lucidum* samples from different provinces of China. With the exception of one sample, the ratio of linoleic to oleic acid in others was below 1.5 [8]. However, the ratio was 1.9 for *G. lucidum* as described by other authors [13]. The ratio of linoleic to oleic acid did not exhibit disparities among different *Ganoderma* species. Additional research is required to ascertain the taxonomic validity of this ratio as an indicator within the *Ganoderma* genus.

Other fatty acids, namely, pentadecanoic acid, myristic acid, hexadecanoic acid, and arachidic acid, were quantified in small amounts, with no significant differences observed between GLU and GLE. Notably, an additional fatty acid, 9-Octadecenoic acid (Z), was exclusively identified in GLE but absent in GLU. 9-Octadecenoic acid (Z) is regarded as a trans isomer of unsaturated fatty acids. Trans isomers of unsaturated fatty acids were not detected in *Ganoderma* strains from Ghana and other wild edible mushrooms [5,33]. This indicated that trans isomers could potentially serve as metabolic biomarkers in GLE.

### 3.3. Bioactive Compounds of GLU and GLE

**Figure 2:**
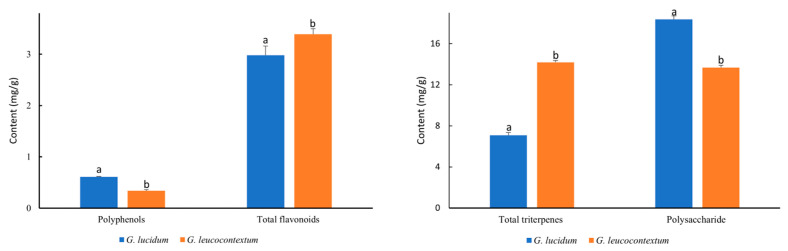
The bioactive constitutes content of GLU and GLE. **Left**: polyphenols and total flavonoids. **Right**: total triterpenes and polysaccharide. Different superscript letters indicate significant differences (*p* < 0.05) between GLU and GLE.

Polysaccharides and triterpenes are the most crucial active fungal components, extensively investigated for their potential health benefits. The polysaccharide and triterpenoid content in GLE was initially reported in our study (Figure 2). GLU exhibited a higher content of water-soluble polysaccharides than GLE (13.68 mg glucose/g). The polysaccharide content in GLU partially aligned with that reported by Taofiq et al. [34], i.e., approximately 18.37 mg glucose/g. Remarkably, the total triterpene content in GLE (14.19 mg oleanolic acid/g) was twice as high as that in GLU (7.08 mg oleanolic acid/g). The triterpenoid content in GLU was consistent with recent findings, where triterpenoid amounts ranged from 5.60 to 10.87 mg/g [24]. The existing literature on the polysaccharides and triterpenes content of *Ganoderma* presents divergent findings. Beyond species variations, the polysaccharide and triterpenoid contents of *Ganoderma* are subject to fluctuations with growth stages, diverse cultivation conditions, and distinct processing methods. Dong et al. noted significant modifications in the bioactive constituents and antioxidant efficacy of *G. lucidum* resulting from different drying methods. The zenith levels of total triterpenoids and polysaccharides in fruiting bodies were determined to occur before maturity, specifically during the stipe elongation stage or the early stage of pileus formation [35]. Skalicka-Woźniak et al. reported the polysaccharide yields of four *G. lucidum* strains cultivated on different wood substrates ranging from 18.45 to 112 mg glucose/g [36]. Our quantification results spanned this range. *Ganoderma* triterpenes, as principal secondary metabolites, were acknowledged for their antitumor, anti-inflammatory, antioxidant, antimicrobial, and blood-fat-reducing properties, extensively employed in complementary cancer therapy [37]. GLE showcased potential therapeutic effects in various pathological conditions.

Flavonoids and polyphenols, active compounds found in plants and fungi, are renowned for their antioxidant, antiproliferative, and anti-inflammatory properties. Phenolic compounds and flavonoids derived from *Ganoderma* have consistently demonstrated excellent antioxidant activities [38]. The polyphenol content was only 0.34 mg GAE/g in GLE, nearly half of that in GLU (0.64 mg GAE/g). However, the total flavonoid content in GLE (3.39 mg QE/g) was significantly higher compared to GLU. This outcome diverged from previously reported findings, where total flavonoids and phenols in *Ganoderma* sp. from Mexico ranged from 38 to 56 mg GAE/g and 0.53 to 0.93 mg QE/g, respectively [39]. The disparity may be closely linked to variations in *Ganoderma* collection and extraction methods, encompassing the choice of extraction solvent and temperature. The total phenol content in extracts obtained from *G. lucidum* fruiting bodies using different solvents decreased in the order of EtOH > MeOH > H2O > Ac > Hex [40]. Kebaili et al. noted that the ethyl acetate extract from *G. lucidum* contained higher flavonoid and phenolic content than chloroform and butanoic extracts [25]. The ethyl acetate extracts also exhibited better antioxidant capacity. Additionally, the ethyl acetate extracts exhibited superior antioxidant capacity, with a significant correlation observed between antioxidant activities and total phenol and flavonoid contents.

### 3.4. Antioxidant Activity of the Ethanol Extract of G. lucidum and G. leucocontextum

**Figure 3:**
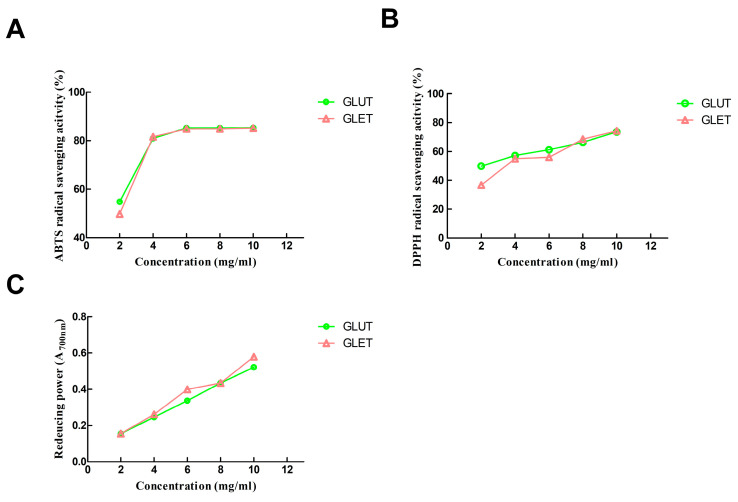
Antioxidant capacity of GLUT and GLET: (**A**) ABTS radical-scavenging assay; (**B**) DPPH radical-scavenging assay; (**C**) ferric reducing power assay.

The effectiveness of *Ganoderma* sp. as a valuable natural antioxidant source has been substantiated, underscoring its potential for nutraceutical development. The bioactive secondary metabolites, including phenolic compounds and triterpenoids, significantly contribute to its antioxidative properties. In this study, the antioxidant activities of ethanol extracts from *G. lucidum* (GLUT) and *G. leucocontextum* (GLET) were assessed using DPPH, ABTS^•+^ free radical scavenging, and FRAP assays. As depicted in Figure 3A, the ABTS^•+^ radical assay, a commonly employed method for assessing antioxidant activity, revealed that both GLUT and GLET exhibited a noteworthy increase in scavenging efficiency at low concentrations (2 mg/mL to 4 mg/mL), followed by a subsequent plateau. The highest inhibition rates of ABTS^•+^ radicals reached 85.31% and 85.18%, respectively. In the DPPH radical scavenging activity, GLUT demonstrated a dose-dependent manner with an IC50 value of 2.234 mg/mL, while GLET exhibited an IC50 value of 3.607 mg/mL (Figure 3B). Initially, GLUT displayed higher activity compared to GLET at concentrations of 2~6 mg/mL, but GLET surpassed GLUT with a further increase in concentration. Regarding the reducing power assay, the absorbance values reflected the reducing ability. GLUT and GLET exhibited similar reducing powers, both displaying a linear increase in the range of 2~10 mg/mL (Figure 3C). Overall, there were no significant differences in ABTS^•+^ scavenging capacity and ferric reducing power between the two extracts.

This study effectively delineated the noteworthy antioxidant attributes of GLUT and GLET in a concentration-dependent manner, consistent with prior research findings [18]. The antioxidant capacity was influenced by the content of bioactive compounds, primarily polyphenols, flavonoids, and triterpenoids, in the extracts from *G. lucidum* [36]. These compounds demonstrated efficient scavenging of diverse free radicals, including DPPH+ and ABTS+, while simultaneously exhibiting proficiency in reducing iron ions. Numerous authors have documented a robust correlation between antioxidant capacities and the content of polyphenolic and flavonoid compounds in *G. lucidum* [18,36,38]. Notably, GLUT displayed a superior DPPH free radical scavenging capacity compared to GLET, potentially attributed to the higher polyphenol content in GLUT. These findings aligned with previous reports on *G. lucidum*, where extracts with high total phenol content exhibited elevated DPPH free radical scavenging activity [39]. Hydroxyl groups and peroxyl radicals, which can induce oxidative damage in vivo, are implicated in various pathological conditions. Our findings suggested that Ganoderma could indeed serve as a valuable source of potent natural antioxidants for therapeutic applications.

### 3.5. The Ethanol Extracts of Ganoderma sp. Suppress the Growth of Tumor Cells via Apoptosis

**Figure 4:**
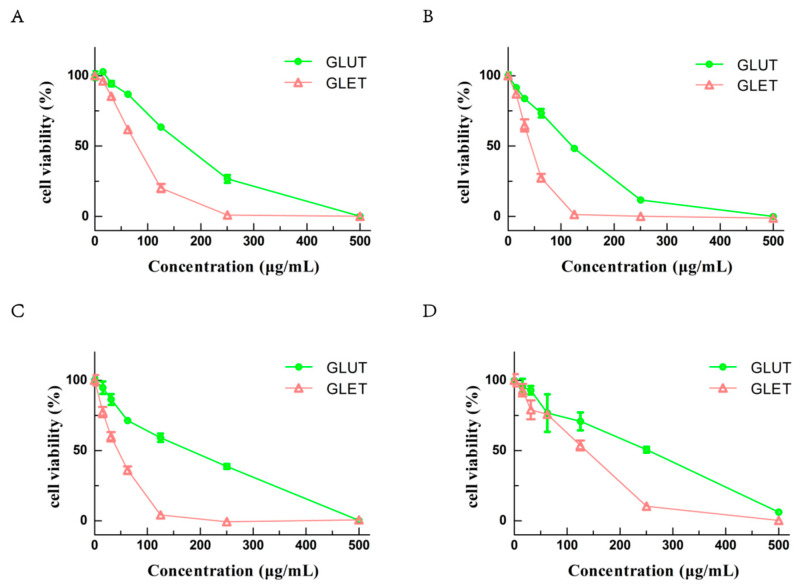
Cell viability of tumor cell after treatment with different concentrations of GLUT and GLET for 48 h: (**A**) A549 cells; (**B**) PC12 cells; (**C**) B16 cells; (**D**) Hela cells.

*G. lucidum* extracts have been employed in folk medicine for years, often recommended as an immune system support supplement in cancer treatment. In our investigation of the effects of GLUT and GLET using the CCK-8 assay on several tumor cells, including A549, PC-12, B16, and Hela cells, both GLUT and GLET demonstrated cytotoxic effects against all tested tumor cells (Figure 4). After 48 h of treatment, cell viability significantly decreased with increasing concentrations of GLUT and GLET. The IC50 values for GLET were 72.76 μg/mL for A549 cells, 19.84 μg/mL for PC12 cells, 19.09 μg/mL for B16 cells, and 55.3 μg/mL for Hela cells. On the other hand, the IC50 values for GLUT were 155.1 μg/mL, 52.42 μg/mL, 70.24 μg/mL, and 97.77 μg/mL for the same cell lines, respectively. PC12 and B16 cells exhibited higher sensitivity to both *Ganoderma* sp. extracts compared to A549 and Hela cells. The antiproliferative activity of various extracts or isolated compounds from *G. lucidum* has been evaluated on various tumor cells in both in vivo and in vitro studies. A previously described study reported a comparable cytotoxic effect of ethanol extracts from the fruiting bodies of *G. lucidum* cultivated on alternative and commercial substrates against HeLa (38.11~109.04 μg/mL) and A549 (26.48~167.08 μg/mL) cells [41].

Our research aimed at comparing the antitumor capabilities of ethanol extracts from two *Ganoderma* species. The inhibitory potential against cancer cells was closely linked to the active ingredients in the ethanol extracts, primarily comprising triterpenes and phenols [42]. Triterpenes, particularly ganoderic acids, were identified as crucial bioactive compounds with potential antitumor properties in ethanol extracts of *Ganoderma*. The triterpenes content in GLE was significantly higher than that in GLU, resulting in a more pronounced antiproliferative activity in GLET. Kolniak-Ostek et al. discovered that the methanolic extract from *G. lucidum*, rich in polyphenols and triterpenoids, exerted a substantial antiproliferative effect on six cancer cell lines in a dose-dependent manner [43]. The ethanolic extract from *G. lucidum* exhibited inhibitory activity on the growth of cervical carcinoma, lung cancer, and colon cancer, with an IC50 value ranging from 120 to 500 μg/mL [44]. Notably, the extract with the lowest concentration of total polyphenols but which was abundant in specific phenolic compounds (Hesperetin and Naringenin) manifested the highest cytotoxic effect. It suggested that the composition and content of particular phenolic compounds in extracts significantly influence their antiproliferative activity.

**Figure 5:**
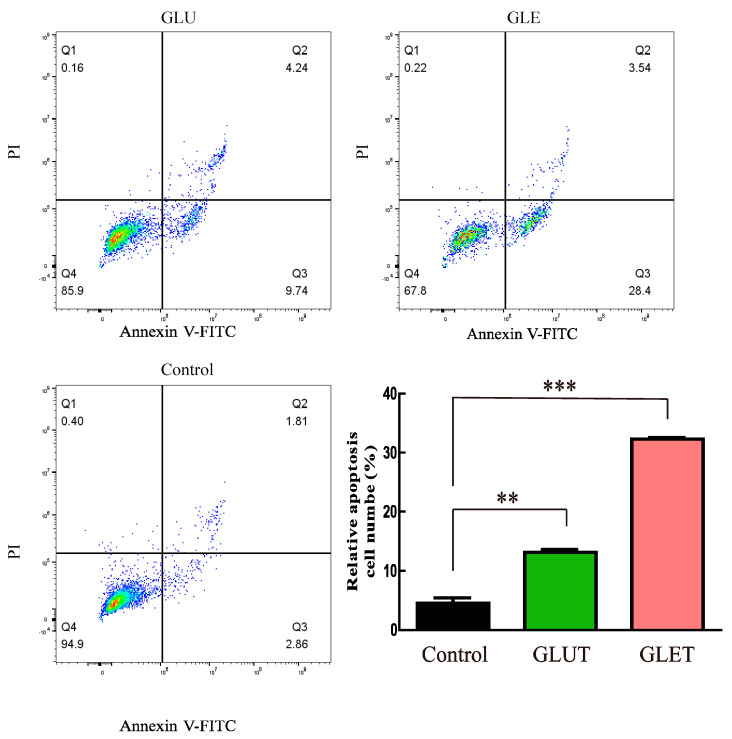
Flow cytometry analysis of PC 12 cells treated by GLUT and GLET. The cells were incubated with GLUT and GLET for 24 h, and the apoptosis was measured via flow cytometry. The number of apoptotic cells in the treatment group was significantly higher than that in the control group. ** *p* < 0.01, *** *p* < 0.05.

**Figure 6:**
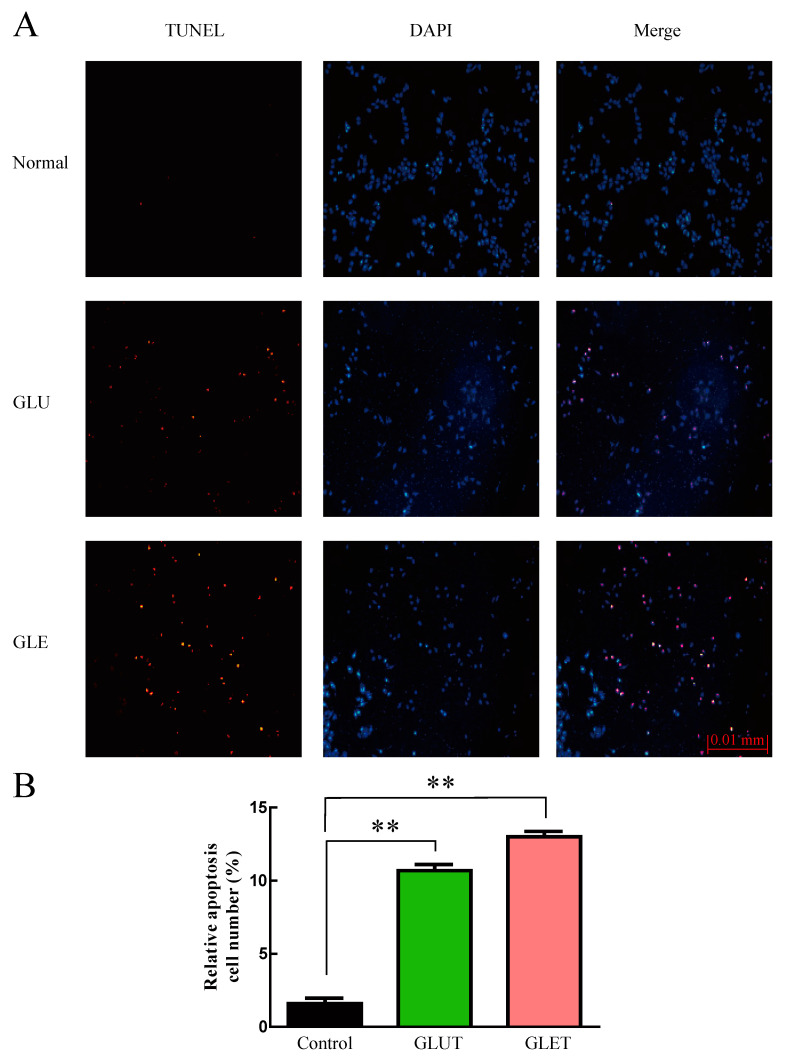
TUNEL staining was performed in PC-12 cells following treatment with GLUT and GLET. The cells were cultured with GLUT and GLET for 24 h and subsequently subjected to TUNEL assay. (**A**) TUNEL and DAPI staining. Apoptosis was determined by TUNEL staining (Red) and the nuclei were stained with DAPI (Blue). (**B**) Quantification of TUNEL positive cells. The number of apoptotic cells was substantially higher in the treatment group compared to the control group. ** *p* < 0.01.

To determine whether GLUT and GLET induced tumor cell apoptosis, a flow cytometry assay was employed using double staining with annexin V-FITC/PI in PC12 cells. As illustrated in Figure 5, the total apoptotic cell population surged in PC12 cells following a 24 h treatment with GLET or GLUT at a concentration of 50 μg/mL. GLET demonstrated the ability to induce 28.4% of cells into early apoptosis (lower right quadrant), which was nearly three times the proapoptotic cells induced by GLUT. Furthermore, TUNEL staining revealed a remarkable increase in the number of apoptotic cells (Red) after treatment with GLET or GLUT at a concentration of 50 μg/mL for 24 h compared with the control cells (Figure 6). There was a significant difference in the apoptotic cells population between the two extracts; it was observed that GLET exhibited a stronger activity in inducing apoptosis in PC12 cells. These findings underscore the potential of GLET as a more potent inducer of apoptosis in tumor cells compared to GLUT.

The reduction in tumor cell viability induced by the treatment of both GLUT and GLET, likely attributable to apoptosis induction, was substantiated through both TUNEL and flow cytometry analyses. It demonstrated that both ethanol extracts significantly promoted tumor cell apoptosis. The antiproliferative activity of the ethanol extract from the fruiting bodies of *G. lucidum* has been previously elucidated, involving multiple mechanisms. The alcohol extract of *G. lucidum* was found to inhibit cell proliferation by up-regulating p21 and down-regulating cyclin D1 [45]. Additionally, it induced cell apoptosis through the overexpression of Bax in human breast cancer cells. The reduction in the expression levels of antiapoptotic proteins and activation of pro-apoptotic proteins were crucial for the induction of apoptosis. Wu et al. described an ethanol-soluble and acidic component (ESAC) from *G. lucidum*, mainly composed of triterpenes, exhibiting antiproliferative effects by inducing DNA damage, initiating G1 cell cycle arrest, and promoting apoptosis in human breast cancer cells [46]. As the primary active ingredient in the ethanol extract of *G. lucidum*, total triterpenes exerted apoptosis-inducing and anticancer activities in vitro and in vivo [47]. A triterpene–farnesyl hydroquinone hybrid (GL22) isolated from the fruiting bodies of *G. leucocontextum* was discovered to decrease mitochondrial membrane potential and induced liver cancer cell apoptosis [18]. These findings suggested that the two ethanol extracts might downregulate the expression of antiapoptotic proteins while increasing or activating the expression of pro-apoptotic proteins, ultimately leading to cell death.

These results indicate that GLUT and GLET have potential therapeutic uses in the prevention and treatment of cancer. Importantly, the extract from *G. lucidum* has shown selective cytotoxicity and did not exhibit toxicity on normal cells [43]. Oral administration of an alcoholic extract from *G. leucocontextum* (GLA) demonstrated no notable toxic effects in both male and female rats during acute and subacute toxicity assessments [48]. Both species, particularly *G. leucocontextum*, hold promise as potential sources of anticancer agents. Nevertheless, the molecular mechanisms of the anticancer activity of GLE are still undergoing further investigation.

## 4. Conclusions

In summary, this study compared the nutritional composition, bioactive compounds, antioxidant capacity, and antitumor activity of *G. lucidum* and *G. leucocontextum*. GLE showed higher levels of protein, ash, flavonoids, and triterpenes compared to GLU, while GLU contained more dietary fiber, polyphenols, and polysaccharides. Both species demonstrated significant antioxidant activity and antitumor effects, with GLE showing higher cytotoxicity. These findings highlight the potential of GLE as a valuable source of antioxidants and anticancer agents, opening avenues for further research on the molecular mechanisms underlying its therapeutic effects.

## Figures and Tables

**Table 1:** Chemical composition of GLU and GLE.

Chemical Composition	*G. lucidum*	*G. leucocontextum*
Total gross fat (g/100 g)	2.02 ± 0.07 ^a^	3.54 ± 0.11 ^b^
Crude protein (g/100 g)	14.0 ± 0.36 ^a^	21.2 ± 0.31 ^b^
Moisture (g/100 g)	9.56 ± 0.43 ^a^	8.93 ± 0.28 ^a^
Ash (g/100 g)	1.20 ± 0.11 ^a^	2.20 ± 0.17 ^b^
Dietary fiber (%)	64.2 ± 2.30 ^a^	58.9 ± 1.30 ^a^
**Mineral** (mg/kg)		
Calcium	1670 ± 0.01 ^a^	683 ± 21.3 ^b^
Magnesium	486 ± 23.0 ^a^	750 ± 28.0 ^b^
Iron	38.5 ± 1.30 ^a^	69.9 ± 3.5 ^b^
Zinc	18.3 ± 1.60 ^a^	42.9 ± 3.9 ^b^
**Amino acid** (mg/g)		
Aspartic acid	8.90 ± 0.35 ^a^	12.6 ± 0.52 ^b^
Threonine	5.00 ± 0.12 ^a^	6.20 ± 0.35 ^b^
Serine	4.80 ± 0.06 ^a^	5.90 ± 0.36 ^b^
Glutamic acid	9.40 ± 0.29 ^a^	17.6 ± 0.43 ^b^
Glycine	4.60 ± 0.40 ^a^	6.30 ± 0.42 ^b^
Alanine	5.30 ± 0.46 ^a^	7.40 ± 0.41 ^b^
Valine	5.20 ± 0.40 ^a^	6.60 ± 0.29 ^b^
Methionine	0.81 ± 0.28 ^a^	1.50 ± 0.23 ^a^
Isoleucine	4.00 ± 0.52 ^a^	4.80 ± 0.35 ^a^
Leucine	6.10 ± 0.33 ^a^	8.40 ± 0.29 ^b^
Tyrosine	1.70 ± 0.44 ^a^	3.10 ± 0.23 ^b^
Phenylalanine	4.00 ± 0.46 ^a^	5.30 ± 0.23 ^a^
Histidine	9.10 ± 0.12 ^a^	12.6 ± 0.46 ^b^
Lysine	4.00 ± 0.11 ^a^	6.60 ± 0.24 ^b^
Argnine	3.10 ± 0.33 ^a^	8.00 ± 0.40 ^b^

Data are mean values ± standard deviation (SD) of duplicate results (the same as blow). Different letters indicate significant differences (*p* < 0.05) between GLU and GLE.

## Data Availability

The original contributions presented in the study are included in the article, further inquiries can be directed to the corresponding author.
